# Polymorphism in Self-Assembled Structures of 9-Anthracene Carboxylic Acid on Ag(111)

**DOI:** 10.3390/ijms13066836

**Published:** 2012-06-05

**Authors:** Chao Lu, Yinying Wei, Erkuang Zhu, Janice E. Reutt-Robey, Bo Xu

**Affiliations:** 1 State Key Laboratory of Metastable Material Science and Technology, Yanshan University, Qinhuangdao 066004, Hebei, China; E-Mails: luchao397@gmail.com (C.L.); zek2008@ysu.edu.cn (E.Z.); 2 Department of Chemistry and Biochemistry, University of Maryland, College Park, MD 20742, USA; E-Mails: wonderland728@gmail.com (Y.W.); rrobey@umd.edu (J.E.R.-R.)

**Keywords:** self-assembly, nanostructures, polymorphism, scanning tunneling microscopy

## Abstract

Surface self-assembly process of 9-anthracene carboxylic acid (AnCA) on Ag(111) was investigated using STM. Depending on the molecular surface density, four spontaneously formed and one annealed AnCA ordered phases were observed, namely a straight belt phase, a zigzag double-belt phase, two simpler dimer phases, and a kagome phase. The two high-density belt phases possess large unit cells on the scale length of 10 nm, which are seldom observed in molecular self-assembled structures. This structural diversity stems from a complicated competition of different interactions of AnCA molecules on metal surface, including intermolecular and molecular-substrate interactions, as well as the steric demand from high molecular surface density.

## 1. Introduction

A comprehensive understanding of the self-assembly process of organic molecules on surface, as well as the pathway to control it, will contribute to the fabrication and optimization of the molecular-based nanostructures for applications [1–5]. These self-assembled nanostructures are usually stabilized by non-covalent forces, such as hydrogen bonding [6–8], metal coordination [9], van der Waals interaction [10,11], and dipole-dipole interaction [12], *etc*. For many applications, well-defined organic thin films are desirable in order to achieve better electrical and optical performances. The growth and structure control of monolayer films are crucial in determining both the morphologies and electronic states of the final films [13–15]. By choosing appropriate building blocks and growth parameters, different adlayer structures can be produced on surfaces [16–24].

The development of the first layer self-assembly structure depends on the subtle balance of intermolecular and molecular-substrate interactions. Systematic studies on the sequence of ordered phases formed in organic molecular adlayer structures with distinct coverage give an insight into the underlying energies [15,16,20,25]. For example, Umbach *et al.* reported a study of tin phthalocyanine (SnPc) on Ag(111), where the high critical nucleation density, continuously evolved structural parameters with coverage, and maximum domain size are attributed to a repulsive intermolecular interaction presenting even at a very high surface molecular density [15]. Moreover, exploring the relationship between surface architecture and bulk crystal structure can also reveal the impacts of substrate and dimensionality on molecular nanostructures [26,27].

Previously, we reported two AnCA adlayer structures on Ag(111) as templates for sequential C_60_ deposition [28]. In this paper, we report the detailed self-assembly process of AnCA on Ag(111) substrate. Four adlayer structures spontaneously formed after molecular deposition are demonstrated. By relating the structural diversity to different interactions and the steric demand on the surface, we reveal that the dominant force controlling AnCA self-assembly on Ag(111) goes from intermolecular interaction at high surface density to molecular-substrate interaction at low surface density. A kagome structure formed after mild annealing at 353 K is also presented.

## 2. Results and Discussion

### 2.1. Results

**Figure 1:**
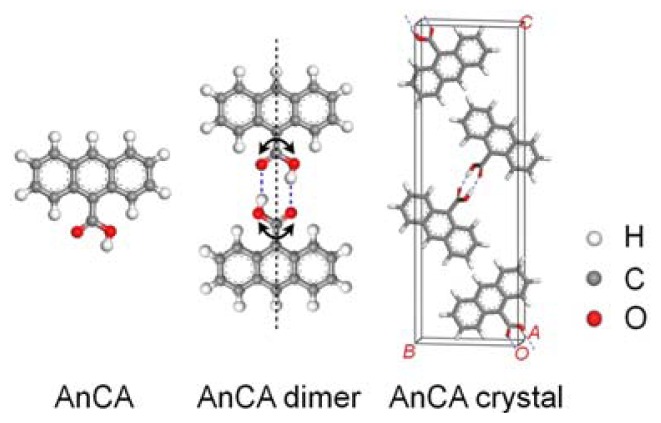
Structures of AnCA molecule, AnCA dimer, and AnCA crystal.

The structures of a free AnCA molecule and an AnCA dimer are illustrated in Figure 1. This molecule has a planar anthracene core and a carboxylic acid functional group which can rotate around the C–C bond after forming head-to-head hydrogen bonds with another AnCA molecule, as shown in Figure 1. The dipole moment of a free AnCA, calculated by Dmol^3^, is about 1.9 D. This dipole is mostly canceled out in the head-to-head AnCA dimer.

After the initial 1 ML AnCA deposition, a series of ordered structures with decreasing surface molecular density was resolved at prolonged time intervals. This is accompanied by a spontaneous desorption of AnCA from the surface at room temperature. For calibration purpose, AnCA molecules were also deposited onto a 0.2 ML C_60_ pre-covered Ag surface. As AnCA is grown through islands nucleation on open Ag terraces, the pre-deposited C_60_ molecules do not influence AnCA self-assembly process. The same calibration method was previously used for a similar molecule, 9-acridine carboxylic acid (ACA) [29]. The structure parameters for each AnCA phase can then be calibrated with respect to the well-known $2\sqrt{3}\times 2\sqrt{3}\;\text{R }30{}^{\circ}$ C_60_ close packed structure on Ag(111). The possibility of multilayer structures of AnCA is ruled out by comparing the topographic height of Ag steps, C_60_, and each AnCA phase. All AnCA structures reported below are of similar height and in the monolayer regime.

**Figure 2:**
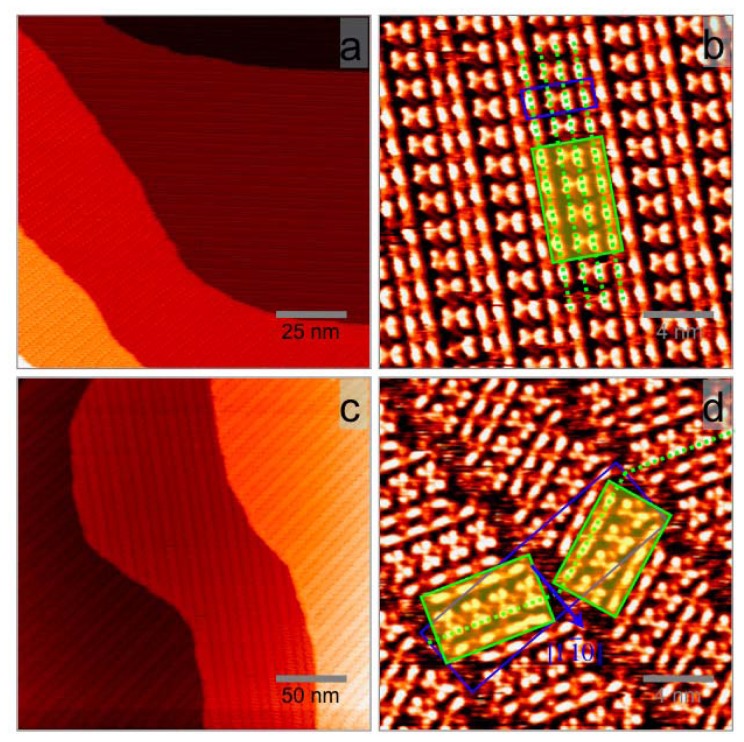
STM images of AnCA Phase I and II with different length scales. (**a**) 125 nm Phase I; (**b**) 20 nm Phase I; (**c**) 250 nm Phase II; (**d**) 20 nm Phase II. The unit cells and building blocks are marked with blue boxes and yellow shadowed boxes, respectively. The green dotted lines emphasize the breakdown of the molecular belt from Phase I to Phase II. The tunneling parameters are (**a**) *V*_T_ = 0.82 V and *I*_T_ = 83 pA; (**b**) *V*_T_ = 0.72 V and *I*_T_ = 85 pA; (**c**) *V*_T_ = 0.88 V and *I*_T_ = 97 pA; and (**d**) *V*_T_ = 0.93 V and *I*_T_ = 85 pA.

The first ordered structure was observed within two hours after the deposition. As shown in Figure 2, it is a parallel belt structure and denoted as Phase I. The molecular belts run along the direction about 8° deviated from Ag[11̄0]. The periodicities along and across the molecular belt direction are *ca.* 1.6 nm and 4.0 nm, respectively. A magnified STM image of this phase is presented in Figure 2b, where each bright feature in the image is assigned to an AnCA dimer based on the molecular dimension. More details about this assignment are presented in the next subsection. A unit cell is marked with the blue box in Figure 2b. The area of the unit cell is *ca.* 6.4 nm^2^, leading to a molecular density of 1.25 molecule per nm^2^, or one AnCA molecule per eleven Ag atoms.

Phase I was not stable and a phase transition was identified during the scanning (supplementary material). The newly formed double-belt structure (Phase II) has the same building block as that of Phase I, illustrated with the yellow shadowed box in Figure 2b,d. In Phase I, all the building blocks are arranged in the same way along the molecular belt direction. While in Phase II, building blocks in the neighboring belts are alternatively orientated, with the short edges of the building blocks rotated away from Ag[11̄0] about 22° in opposite directions and shifted by half the block size along Ag[11̄0]. Phase II, possessing a glide-reflection symmetry with respect to Ag[11̄0], can be looked as a racemic mixture of two mirror domains of Phase I. It has an exceptionally large unit cell of *ca.* 60 nm^2^ in area, emphasized with the large blue box in Figure 2d. The surface crystallinity is remarkably good. We also notice from STM images the areas between neighboring belts are noisy, which are attributed to 2 dimensional (2d) gas AnCA molecules with density lower than that of the above-mentioned building blocks (1.25 molecule per nm^2^). The molecular density of Phase II thus ranges between 1.06–1.25 molecule per nm^2^ depending on the mobile molecular density. The density minimum is determined by ignoring the mobile molecules.

**Figure 3:**
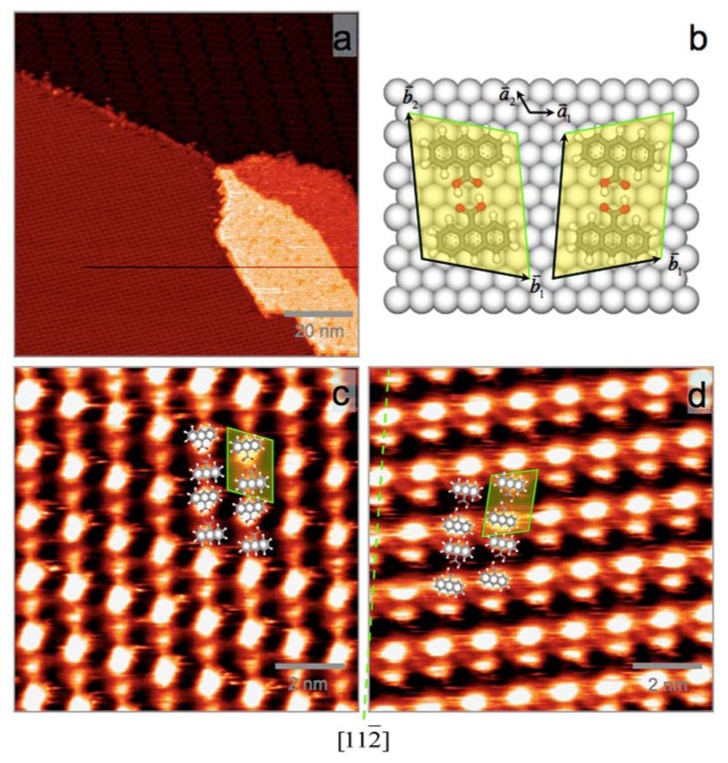
(**a**) Large-scale STM image of AnCA Phase III (lower left), along with C_60_ (lower right) and AnCA Phase II (upper right); (**b**) Molecular packing model of two enantiomeric domains; (**c**) and (**d**) STM images of two enantiomeric domains symmetric with respect to Ag[112̄] (the green dotted line). Two unit cells corresponding to those in (**b**) are marked with yellow shadowed boxes in (**c**) and (**d**). The tunneling parameters are (**a**) *V*_T_ = 0.85 V and *I*_T_ = 87 pA; (**c**) *V*_T_ = 0.93 V and *I*_T_ = 95 pA; and (**d**) *V*_T_ = 0.84 V and *I*_T_ = 105 pA.

After more AnCA molecules desorbed from the surface, a new superstructure (Phase III) was formed on Ag(111). As shown in Figure 3, it is a simpler ordered AnCA structure where individual molecules can be resolved. This phase is commensurate with Ag(111) surface with lattice vectors off the high symmetry directions of Ag surface. As a result, there are two enantiomeric domains coexistence on the surface, indexed as (4 −1, 3 7) and (5 1, 4 7) in matrix notation with respected to Ag(111) lattice vectors. STM images overlapped with the suggested molecular packing models are shown in Figure 3c,d for these enantiomeric domains, which are mirror-symmetric with respect to Ag[112̄]. Unit cells consisting of one dimer are marked with yellow shadowed boxes in Figure 3c,d, where each spot corresponds to a single AnCA molecule. The shape and brightness difference of the spots observed here can be attributed to nonequivalent AnCA orientations inside the dimer. Similar difference has been previously observed [30]. The molecular density is calculated to be 0.90 molecule per nm^2^, or one AnCA molecule per 15.4 Ag atoms. Molecular packing models are presented in Figure 3b.

**Figure 4:**
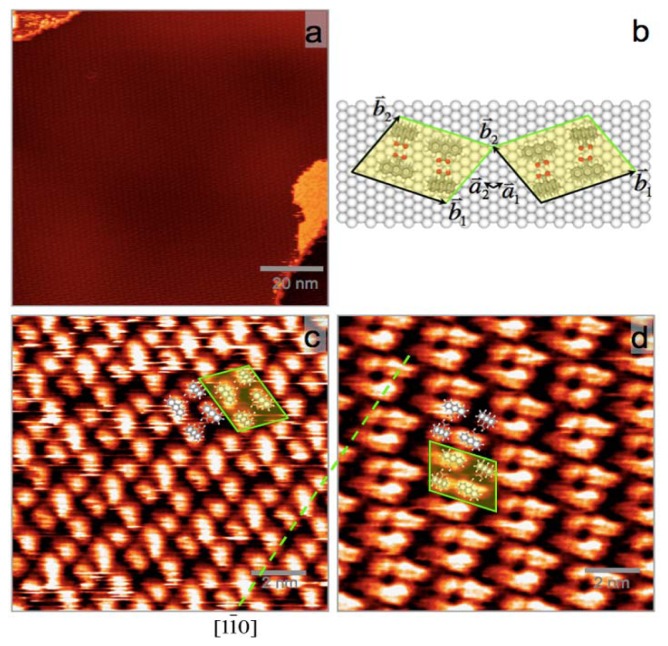
(**a**) Large-scale STM image of AnCA Phase IV; (**b**) Molecular packing models of two enantiomeric domains; (**c**) and (**d**) STM images of two enantiomeric domains symmetric with respect to Ag[11̄0] (the green dotted line). Two unit cells corresponding to those in (**b**) are marked with yellow shadowed boxes in (**c**) and (**d**). The tunneling parameters are (**a**) *V*_T_ = 1.05 V and *I*_T_ = 80 pA; (**c**) *V*_T_ = 0.97 V and *I*_T_ = 104 pA; and (**d**) *V*_T_ = 0.98 V and *I*_T_ = 103 pA.

Phase III can further evolve to another structure (Phase IV) after more AnCA molecules desorbed from the surface, as shown in Figure 4. Phase IV is also commensurate with Ag(111) surface and sorted into two enantiomeric domains indexed as (2 −8, 8 3) and (8 −2, 3 8), respectively. STM images of the enantiomeric domains, mirror-symmetric with respect to Ag[11̄0] direction, are shown in Figure 4c,d overlapped with the suggested molecular packing models. Unit cells consisting of two dimers are marked with yellow shadowed boxes in Figure 4c,d. The molecular packing density in Phase IV is 0.79 molecule per nm^2^, or one AnCA molecule per 17.6 Ag atoms. Molecular packing models are presented in Figure 4b.

**Figure 5:**
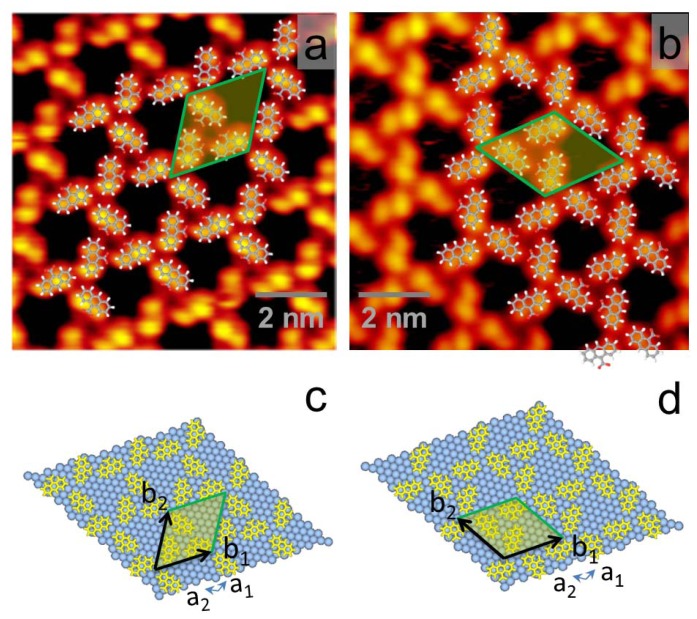
(**a**) and (**b**) STM images of two enantiomeric domains of Phase V symmetric with respect to Ag ; (**c**) and (**d**) Molecular packing models of two enantiomeric domains. The unit cells are marked with yellow shadowed boxes. The tunneling parameters are *V*_T_ = 1.5 V and *I*_T_ = 120 pA for both STM images.

Phase IV is stable at room temperature. After annealed it at 353 K for 30 min, most AnCA molecules are desorbed from the surface. Occasionally, an AnCA kagome structure (Phase V) is observed, which is usually smaller than 20 nm in size and sorted into two enantiomeric domains indexed as (4 −5, 9 4) and (5 −4, 4 9). STM images overlapped with the suggested molecular packing models are shown in Figure 5a,b for these enantiomeric domains, which are mirror-symmetric with respect to Ag[112̄]. The molecular density is 0.68 molecule per nm^2^, or one AnCA molecule per 20.4 Ag atoms. Molecular packing models are presented in Figure 5c,d.

### 2.2. Discussion

#### 2.2.1. Adlayer *vs*. Crystal

AnCA molecular crystal has a *P*2_1_/*n* symmetry with *a* = 0.39 nm, *b* = 0.94 nm, and *c* = 2.9 nm, (see Figure 1 for the structure) [31]. This crystal structure can be rationalized as molecular dimer planes stacked along the [100] direction. Inside each plane, coplanar AnCA dimers are organized into herringbone configuration with anthracene cores parallel to the (100) plane. Despite its simple crystal structure, AnCA self-assembly on Ag(111) displays versatile phase behaviors: Five adlayer structures (4 spontaneously formed and 1 annealed) were observed with distinct molecular surface density.

For the four spontaneously formed AnCA structures on Ag(111) as well as the bulk crystal, the primary building block is AnCA dimer formed through hydrogen bonding of two AnCA molecules. Our DFT calculations suggest an isolated AnCA molecule bears prominent electrical dipole and quadrupole moments, 1.9 D and 22 D·Å, respectively. After the formation of dimer, the molecular dipole moment is mostly cancelled out while the total quadrupole moment is enhanced, and two AnCA molecules can be stabilized through two O···H–O hydrogen bonds with an energy gain of 29 kJ/mol. The dihedral angle (DA) between two anthracene cores in an isolated AnCA dimer is 79° and the length of the hydrogen bond is 1.67 Å. In bulk crystal, the π–π interactions between AnCA molecules in neighboring (100) planes provide strong stabilization energy, which is maximized by stacking dimers parallel along [100] direction. Such kind of crystal structure is popular in aromatic molecules [32]. However, these stacking π–π intermolecular interactions are absent when AnCA dimers adsorbed on Ag substrate (this may presumably contribute to the spontaneous desorption process of AnCA), leading to varied DA depending on surface molecular density. The observed structural diversity on Ag thus stems from the competition of different interactions of AnCA molecules on metal surface, including intermolecular and molecular-substrate interactions, as well as the steric demand due to different molecular surface densities.

#### 2.2.2. Evolution of AnCA Structures

The surface structure evolves with time on Ag(111) surface. Phase I is the dominant structure on surface within two hours after the deposition, Phase II is prevailing within 10 hours, and then Phase III within 24 hours. Phase IV, the most stable one, is the only structure on the surface several days later. The phase transition processes were identified during STM measurements (supplementary material). If we leave a freshly deposited sample in vacuum and take STM measurements only at the specific times mentioned above, corresponding phase would prevail on the surface. The structural evolution observed in the experiment is thus attributed to a spontaneous process along with AnCA desorption.

Table 1 summarizes the four spontaneous AnCA self-assembly structures as well as the annealed one. During the experiments, three phase transitions were observed. These phase transitions occur with a decrease in surface molecular density, indicating a structural instability for at least the first three phases. We noted that ordered structures are difficult to form if the initially deposited AnCA is less than 0.7 ML, suggesting a high mobility for AnCA on Ag surface. The high molecular nucleation density could be attributed to a dominant substrate-mediated repulsive intermolecular interaction as reported in SnPc/Ag(111) system [15].

It is difficult to suggest molecular packing models for Phase I and II due to the limited STM resolution. Tentatively, we assign the bright feature in Figure 2b,c to one AnCA dimer based on the following considerations. From the perspective of surface molecular density, the assignment of the feature to a single molecule would result in a density (Phase I, 1 AnCA per 22 Ag atoms) less than those of Phase III (1 AnCA per 15.4 Ag atoms) and Phase IV (1 AnCA per 17.6 Ag atoms), which contradicts the molecular desorption process we have observed in the experiments. Our DFT calculation suggests an AnCA dimer in the gas phase bear a substantial twist angle of 79° between two anthracene cores. A tilted orientation of the constituting AnCA molecules on Ag is thus expected, leading to high molecular packing densities. Our previous studies of ACA also suggested a tilted molecular orientation on Ag(111) for both the chain phase and the dimer phase [18]. The assignment of the feature to a dimer leads to one AnCA molecule per 11 Ag atoms in Phase I, which is comparable to ACA/Ag(111) system.

The AnCA dimers in Phase I are uniformly spaced along the molecular belt direction but unequally spaced across the belt, as shown in Figure 2b. The two center rows inside the unit cell are closer to each other (forming butterfly-shaped features) and the side rows are equally spaced between the center rows. Although the exact packing configuration cannot be determined, it is plausible to think that AnCA dimers in each row adopt different tilt configurations with respect to Ag surface considering the relatively small area to accommodate an AnCA dimer. The different spacings between the dimers across the molecular belt direction render different couplings between the anthracene cores of adjacent dimer rows. This picture can be rationalized as following: inside the unit cell, a favorable coupling configuration between two center dimer rows (with shorter distance in between) may lead to unfavorable coupling configuration of the side dimer rows, resulting in bigger spacing for the side dimer rows. Similar situation was observed elsewhere [33].

The dimer arrangement characters in Phase I are reserved in Phase II. However, the original straight belt structure is broken into a zigzag structure. In Phase II, half of the molecules form building blocks with the same direction as the original Phase I and assemble into one belt species. The other half form building blocks mirror symmetric with respect to the original one about Ag[11̄0] direction and assemble into another belt species. During this phase transition process, the surface molecular density is decreased and 2d molecular gas (noisy areas) is introduced between neighboring molecular belts. These gas phase molecules increase the entropy of the system and lower the system energy, thus effectively reduce the stress presented in the film. The large unit cells as well as the long range ordering suggest an intricate interaction scenario. Possible factors causing these periodical packing faults include Ag surface effects, high order multipole dispersion forces, and molecular steric demand due to high surface density.

It is well known that on close packed metal surfaces (hcp(0001) and fcc(111)), strain-induced structures can cause misfit dislocations. Such dislocations in elemental systems usually generate two types of structures: striped patterns and triangular patterns [34,35]. Among the stripe patterns, Au(111) herringbone reconstruction is intensively studied [36,37]. For molecular self-assembled films, such strain relief processes were also observed. For example, the self-assembled structure of chlorine zinc phthalocyanine (ZnPcCl_8_) on Ag(111) shows stripe patterns with periodical faults due to the mismatch between the equilibrium molecular unit cell and Ag substrate [38], and titanyl phthalocyanine (TiOPc) molecules on Ag(111) assemble into periodical triangular pattern when deposition flux is high [20]. Both of them are kinetically accessed and thermally metastable structures. For AnCA, the sequential formation and breakdown of Phase I and II may similarly reflect a strain release process. One common thing for ZnPcCl_8_, TiOPc, and AnCA on Ag(111) is the relatively strong intermolecular interaction at high surface density, which is comparable to or even stronger than the adsorbate-substrate interaction. At highest surface density, AnCA molecules form Phase I. The stress, partially relieved through uneven dimer spacing across the belt direction, is presented along the belt direction. This structure is energetically unstable and consecutively breaks down along the belt direction (dotted green line in Figure 2b,d) into AnCA Phase II by introducing periodical packing faults. Details of this phase transition need to be explored by a low temperature STM measurement as well as theoretic calculations.

The coverage dependent AnCA growth experiments show that no ordered structure can be observed if the initial AnCA coverage is lower than 0.7 ML. Pure Phase IV and Phase III structures can be prepared on Ag surface with *ca.* 0.7 ML and 0.8 ML AnCA coverage, respectively. However, we do not observe the reversed phase transitions by introducing extra AnCA molecules to the already formed low-density phases. This result further corroborates that the high-density phases (Phase I and II) are metastable structures and can only be accessed kinetically.

In thermodynamically metastable Phase I and II, AnCA molecules are highly tilted on the surface to accommodate the high molecular surface density. The intermolecular interaction is dominant in these phases. However, they are kinetically trapped structures and not stable at room temperature. As partial AnCA molecules desorb from the surface, interaction between AnCA and Ag substrate becomes dominant. Molecules tend to occupy more space on Ag substrate to gain more adsorption energy. Compared with Phase I, the packing densities for Phase III and Phase IV decrease by 28% and 37%, respectively. The building units (AnCA dimers) are now clearly resolved with STM measurements for low density phases. The reduced surface density and improved STM resolution suggest smaller tilting angles of molecules on Ag surface. A larger overlapping between conjugate π electrons of the anthracene core and metal surface is thus expected, leading to the commensurate Phase III and IV and improved structural stability of AnCA on Ag surface. The annealed kagome structure (Phase V) provides a further proof of this argument. In this phase, the carboxyl groups deprotonate during annealing [39,40], and weak hydrogen bonds form between oxygen atoms and ring hydrogen atoms (O···H–C). The flat-lying molecular configuration yields the lowest surface density, ensures the strongest AnCA-Ag interaction, and results in the best STM resolution.

#### 2.2.3. Comparison with ACA

It is interesting to compare the self-assembly structures of ACA and AnCA on Ag(111). Only one ACA dimer phase was observed on the open Ag terrace while four distinct AnCA dimer phases (Phase I–IV) were observed. Previous studies suggested that when heterocyclic molecules adsorbed on metal surfaces, the orientation of the adsorbate reflects a balance between π-substrate bonding and σ-substrate bonding [41,42]. As coverage increases, the ring plane tilts up from the surface to enhance the interaction between the lone pair electrons and the substrate. In ACA dimer phase, this enhanced interaction between nitrogen lone pair electrons and Ag surface at high coverage would impede the twist flexibility of individual molecule inside ACA dimer and result in a single dimer phase. In contrast, AnCA dimers (without such effect) can twist flexibly with increasing coverage and result in a series of dimer phases with distinct coverage. In addition, as suggested in the reflection adsorption infrared spectroscopy measurements (RAIRS), AnCA molecules are bound into dimers on Ag surface at very low coverage. Increasing AnCA coverage, the dimer configuration is maintained with an increasing molecular tilt angle on Ag surface [43]. ACA molecules, however, are preferentially linked by head-to-tail hydrogen bonds (O–H···N) at low and intermediate coverage, forming the chain phase. Only at relatively high coverage, ACA dimers emerge due to the steric effect.

## 3. Experimental Section

Experiments were performed with an SPECS Aarhus STM150 system operated at room temperature (300 K) with a base pressure better than 1 × 10^−10^ mbar. All reported images were collected in constant current mode. The substrates used in our experiments were 500 nm thick Ag(111) single crystal films, prepared by physical vapor deposition of Ag on mica supports [44]. The films were sputtered with Argon ion (1000 V, 0.5 μA) and subsequently annealed to 800 K, leading to atomically smooth Ag(111) surfaces with terrace widths larger than 100 nm. AnCA and C_60_ (Aldrich) were deposited from separate Knudsen cells (CREATEC) 20 cm away from the substrate at 350 K and 600 K, respectively. The substrate was held at room temperature and the pressure in the preparation chamber was maintained around 1 × 10^−9^ mbar during the deposition. A nearby quartz crystal microbalance was used to estimate the deposition rate in combination with direct STM measurements. The deposition rates for AnCA and C_60_ (for calibration purpose) are 0.07 ML/min and 0.05 ML/min, respectively. After the formation of Phase IV, the sample is subject to annealing at 353 K for 30 min to investigate its thermal stability. Here, one monolayer (ML) of C_60_ is defined as one C_60_ molecule per twelve Ag surface atoms, and one monolayer of AnCA is defined as one AnCA molecule per eleven Ag surface atoms corresponding to AnCA Phase I structure.

## 4. Conclusions

The self-assembly process of AnCA molecules on Ag surface was investigated. A series of ordered structures is formed depending on the molecular surface density. Among the spontaneously formed structures, Phase IV is thermodynamically most stable and undergoes no further phase transition without annealing. Tentative explanations, based on the competition of intermolecular interaction, molecular-substrate interaction, as well as the steric demand due to molecular surface density, are presented for this structural diversity. Structures such as Phases I and II with large lattice parameters are of particular interest in many applications such as photovoltaic cells composed of segregated donor-acceptor domains. The periodical dislocations in molecular thin films supported by metal substrates provide a potential method to regulate a second molecular species. Moreover, the high nucleation density of AnCA ensures a homogeneous 2d gas filling of the surface terraces, which would maximize the structural domain size, limited only by the terrace width as demonstrated in the large-scale STM images. By choosing materials with high critical nucleation density and generating highly homogeneous 2d molecular gas on surface, large-scale ordered domains with single structure could be achieved.

## Supplementary Information



## Figures and Tables

**Table 1:** Lattice parameters of five AnCA phases.

Structure	Phase I	Phase II	Phase III	Phase IV	Phase V
a (nm)	1.6	4.3	1.32	2.02	2.26
b (nm)	4.0	14.0	1.76	2.65	2.26
α (°)	~90	~90	74	71	60
Density (mol/nm^2^)	1.25	1.06~1.25	0.9	0.79	0.68
