# Supplementary material for: Polymorphism in Self-Assembled Structures of 9-Anthracene Carboxylic Acid on Ag(111)
Source: Int J Mol Sci. 2012 Jun 5;13(6):6836–48. doi: 10.3390/ijms13066836 (PMC3397498; doi:10.3390/ijms13066836)
Supplement: Supplementary file 1 [file ijms-13-06836-s001.pdf]

Polymorphism in Self-Assembled Structures of 9-Anthracene Carboxylic Acid on Ag(111)

Supplementary Information

1. Phase Transition from Phase I to Phase II

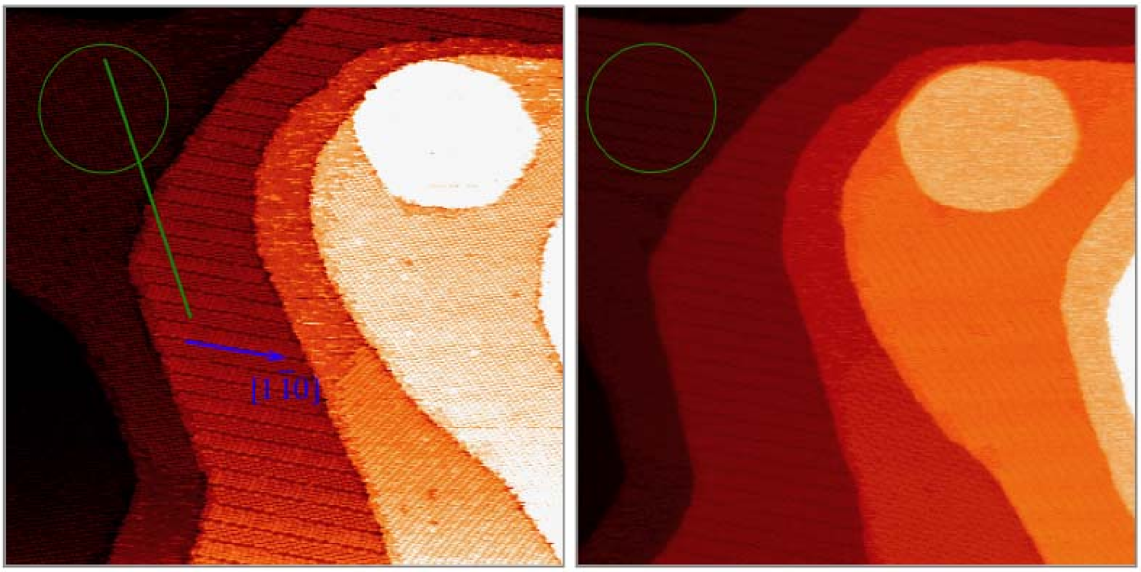

In these two consecutively scanned STM images, the initial AnCA Phase I structure (left image) experienced a phase transition into Phase II structure (right image) as emphasized with the green circles in the images. The green line indicates, in Phase II, half of the molecules form building blocks with the same orientation as Phase I and assemble into one belt species. The other half form building blocks, which are mirror symmetric with respect to the original one about Ag$[1\bar{1}0]$ direction, assemble into another belt species. The acquisition time for both image is *ca.* one minute, and the right image is scanned just after the left one. The STM images are both 250 nm in width.

2. Phase Transition from Phase II to Phase III

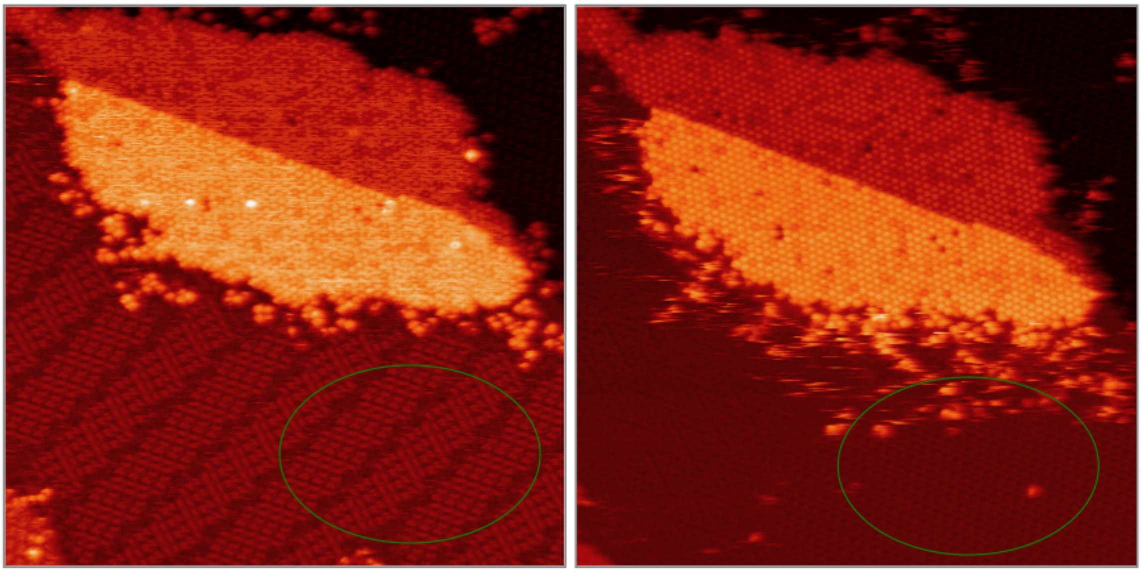

In these two consecutively scanned STM images, the initial AnCA Phase II structure (left image) underwent a phase transition into Phase III structure (right image) as emphasized with the green circles in the images. The acquisition time for both image is *ca.* 50 seconds, and the right image is scanned just after the left one. Both images are 60 nm in width.

3. Phase Transition from Phase III to Phase IV

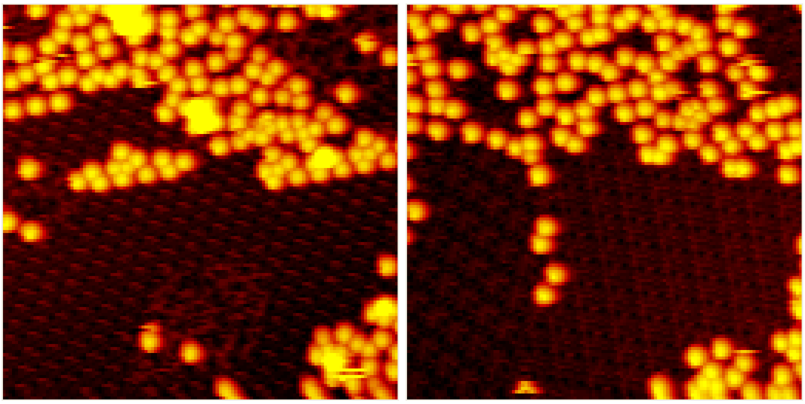

In these two STM images, the initial AnCA Phase III structure (left image) underwent a phase transition into Phase IV structure (right image). The acquisition time for both image is *ca.* 4 seconds, and the right image is scanned 500 seconds later than the left one. Both images are 30 nm in width.
